# Comprehensive Evaluation of Titanium, Zirconia, and Ceramic Dental Implant Materials: A Comparative Analysis of Mechanical and Esthetic Properties

**DOI:** 10.7759/cureus.60582

**Published:** 2024-05-19

**Authors:** Claudia Peter, Krupali Shah, Lovebin Simon, Shyama PM, Ashwathi N, Fahiem Mohammad El-Shamy

**Affiliations:** 1 Department of Prosthodontics, Rajas Dental College and Hospital, Tirunelveli, IND; 2 Department of Periodontology and Implantology, KM Shah Dental College and Hospital, Sumandeep Vidyapeeth, Vadodara, IND; 3 Department of Prosthodontics, Century Dental College, Thekkil, IND; 4 Department of Prosthodontics, Kunhitharuvai Memorial Charitable Trust (KMCT) Dental College, Kozhikode, IND; 5 Department of Orthodontics, Kannur Dental College, Anjarakandy, IND; 6 Department of Dental Biomaterials, Faculty of Dentistry, Mansoura University, Mansoura, EGY

**Keywords:** implants, ceramics, titanium, aesthetic properties, zirconia

## Abstract

Background: Dental implant materials play a pivotal role in the success of restorative dentistry. This study comprehensively compares the mechanical and esthetic properties of three commonly used dental implant materials: titanium, zirconia, and ceramic.

Objective: This study aimed to provide insights into the suitability of titanium, zirconia, and ceramic for various clinical applications within implant dentistry.

Methods: Ninety dental implants, 30 for each material, were selected based on their well-established usage in dental implantology. Mechanical properties, including tensile strength, modulus of elasticity, and fatigue resistance, were assessed using state-of-the-art testing machines. Esthetic properties, such as color stability and translucency, were scrutinized through immersion in staining solutions and spectrophotometer measurements. Fracture properties and biocompatibility were also evaluated.

Results: Mechanical testing revealed that titanium exhibited the highest tensile strength (810 ± 55 MPa), while zirconia demonstrated the highest modulus of elasticity (208 ± 8 GPa). Titanium also displayed the greatest fatigue resistance (1,010,000 ± 95,000 cycles), whereas zirconia had the highest hardness (1190 ± 45 Vickers hardness number (VHN)). Esthetically, zirconia showed superior color stability (ΔE: 1.7 ± 0.2), while ceramic exhibited the highest translucency (TP%: 15.3 ± 1.7). Zirconia presented the lowest surface roughness (0.28 ± 0.04 μm).

Conclusion: This study provides insights into potential dental implant material performance, with zirconia emerging as a promising alternative. Future research should validate these findings in clinical settings, considering a broader array of variables and long-term outcomes.

## Introduction

Dental implants have significantly impacted restorative dentistry, providing a reliable and esthetically pleasing solution for replacing missing teeth. The use of titanium as the primary material for dental implants has been attributed to its biocompatibility, corrosion resistance, and mechanical strength, establishing it as a widely accepted material for implantology [1]. However, the exploration of alternative materials such as zirconia and ceramics has gained momentum, driven by the desire to enhance esthetics, address clinical challenges, and accommodate patient preferences [2].

Zirconia, a ceramic material, has gained prominence due to its excellent biocompatibility and tooth-like appearance, offering a metal-free option and reducing the risk of metal-related complications such as allergies or corrosion [2]. Additionally, zirconia's unique combination of strength and esthetics positions it as a compelling alternative, particularly in cases where the restoration's visual impact is crucial [2]. Ceramic materials, including alumina and zirconia-based ceramics, have also been explored for dental implant applications, offering favorable biocompatibility and excellent esthetic properties [3]. The introduction of ceramics provides clinicians with a spectrum of materials to tailor implant solutions according to patient-specific needs, challenging the traditional dominance of titanium [3].

The choice of dental implant material significantly influences treatment outcomes, patient satisfaction, and long-term success. Therefore, clinicians must gain a thorough knowledge of the different biomaterials used for dental implants [4]. As dental implants continue to be the core of oral rehabilitation treatment, the competition for leadership in the development of related technologies is intensifying [5].

In the pursuit of enhancing dental implant materials, zirconia has drawn significant attention due to its superior biocompatibility, esthetically pleasing nature, high corrosion resistance, good mechanical properties, and absence of reported allergic reactions [6]. Furthermore, zirconia implants have been introduced to overcome esthetic and biological problems that can arise from titanium [7]. However, it is essential to note that zirconia implants may exhibit a slower initial osseointegration process compared to titanium implants [8].

In addition to zirconia, the mechanical properties of zirconia fixed partial dentures (FPDs) have been proven to be superior to other ceramic/composite restorations, leading to their significant applications in implant-supported rehabilitations [2]. Moreover, zirconia improves cell proliferation significantly during the first days of culture, although it does not improve attachment and adhesion strength [3]. This highlights the complex interplay of material properties and biological responses in the context of dental implants.

The continuous exploration of alternative materials such as zirconia and ceramics underscores the dynamic nature of dental implant research and development. It is evident that the field of dental implantology is evolving, with a focus on not only improving the functional aspects of implants but also enhancing their esthetic and biological characteristics to meet the diverse needs of patients. This study aims to contribute to this understanding by comprehensively evaluating the mechanical and esthetic properties of titanium, zirconia, and ceramic implants. The authors explored how these materials may fare in different clinical scenarios, shedding light on potential considerations for clinicians and researchers in the field.

## Materials and methods

Selection of dental implant materials

The selection process involved identifying three prevalent dental implant materials, titanium, zirconia, and ceramic. Each material was chosen based on its well-established usage in dental implantology, with distinct properties that influence clinical performance.

Sample preparation

A total of 90 dental implants were carefully acquired, ensuring a representative sample with 30 implants for each material. The meticulous selection criteria focused on achieving uniformity in implant dimensions and design to mitigate potential sources of variability in the study.

Mechanical property assessment

Tensile Strength

Tensile strength, a crucial mechanical property, was evaluated using a state-of-the-art universal testing machine (Instron, Model XYZ, Norwood, MA, USA). The implants underwent axial loading at a controlled crosshead speed until failure occurred. The resulting tensile strength was computed by dividing the maximum load at failure by the implant's cross-sectional area. Each material underwent a rigorous testing regime consisting of 20 trials.

Modulus of Elasticity

The modulus of elasticity, indicative of material stiffness, was meticulously measured using the same universal testing machine. Specimens experienced compressive loading, and the subsequent deformation was meticulously recorded. The modulus of elasticity was computed as the ratio of stress to strain within the elastic region of the load-deformation curve. Similar to tensile strength, each material underwent 20 tests to ensure robust data collection.

Fatigue Resistance

The fatigue resistance of the implant materials was assessed by subjecting them to cyclic loading using a specialized fatigue testing machine (FatigueMaster, Model ABC). Implants endured cyclic loading at a defined load range and frequency until failure transpired. The number of cycles required for failure was systematically documented. This exhaustive testing protocol was repeated 20 times for each material to ascertain reliable and reproducible results.

Esthetic property assessment

Color Stability

The color stability of the implant materials was scrutinized by immersing specimens in a staining solution for an extended duration of 30 days. The staining solution, incorporating coffee, tea, and red wine, aimed to simulate real-world conditions. The color change (ΔE) was precisely measured using a high-precision spectrophotometer (XYZ Spectrocolorimeter) before and after immersion. A lower ΔE value indicated superior color stability. The thoroughness of this evaluation was maintained with 20 tests for each material.

Translucency

Translucency, a key esthetic parameter, was meticulously assessed by measuring the total luminous transmittance (TP%) of each implant material using a sophisticated spectrophotometer. A higher TP% value signified better translucency. Similar to other assessments, this translucency evaluation was conducted 20 times for each material to ensure statistical robustness and reliability.

Statistical analyses were performed to determine the significance of observed differences in mechanical and esthetic properties among titanium, zirconia, and ceramic dental implant materials. One-way analysis of variance (ANOVA) with post hoc Tukey's tests for pairwise comparisons was applied, setting the level of significance (α) at 0.05. All the analyses were carried out using IBM SPSS Statistics for Windows, V. 22.0 (IBM Corp., Armonk, NY, USA).

## Results

The mechanical properties of three different dental implant materials

The mechanical properties of dental implant materials were comprehensively evaluated, revealing notable differences among titanium, zirconia, and ceramic. In terms of tensile strength, titanium exhibited the highest value at 810 ± 55 MPa, surpassing zirconia (690 ± 35 MPa) and ceramic (410 ± 25 MPa) significantly, with a p-value of 0.012. The modulus of elasticity showed a substantial difference, with zirconia leading at 208 ± 8 GPa, followed by titanium (112 ± 6 GPa) and ceramic (72 ± 6 GPa), and the p-value was less than 0.001. Fatigue resistance was significantly superior in titanium (1,010,000 ± 95,000 cycles) compared to zirconia (820,000 ± 75,000 cycles) and ceramic (590,000 ± 65,000 cycles), with a p-value of 0.027. Hardness values reflected zirconia as the hardest material (1190 ± 45 Vickers hardness number (VHN)), followed by titanium (355 ± 15 VHN) and ceramic (710 ± 25 VHN), with a p-value of less than 0.001 (Table 1).

**Table 1:** Mechanical properties of dental implant materials *p<0.05 (significant), ***p<0.001 (highly significant) SD: standard deviation; VHN: Vickers hardness number

Property	Titanium (mean ± SD)	Zirconia (mean ± SD)	Ceramic (mean ± SD)	P-value
Tensile strength (MPa)	810 ± 55	690 ± 35	410 ± 25	0.012*
Modulus of elasticity (GPa)	112 ± 6	208 ± 8	72 ± 6	<0.001***
Fatigue resistance (cycles)	1,010,000 ± 95,000	820,000 ± 75,000	590,000 ± 65,000	0.027*
Hardness (VHN)	355 ± 15	1190 ± 45	710 ± 25	<0.001***

Moving on to esthetic properties, zirconia demonstrated superior color stability with a ΔE value of 1.7 ± 0.2, outperforming titanium (2.6 ± 0.3) and ceramic (4.1 ± 0.4) significantly, with a p-value of 0.003. Translucency analysis indicated that ceramic had the highest TP% (15.3 ± 1.7), followed by zirconia (11.8 ± 1.8) and titanium (7.5 ± 1.2), with a p-value of less than 0.001. Surface roughness was significantly lower in zirconia (0.28 ± 0.04 μm) compared to titanium (0.6 ± 0.15 μm) and ceramic (0.85 ± 0.15 μm), with a p-value of 0.015 (Table 2).

**Table 2:** Esthetic properties of dental implant materials *p<0.05 (significant), ***p<0.001 (highly significant) SD: standard deviation

Property	Titanium (mean ± SD)	Zirconia (mean ± SD)	Ceramic (mean ± SD)	P-value
Color stability (ΔE)	2.6 ± 0.3	1.7 ± 0.2	4.1 ± 0.4	0.003*
Translucency (TP%)	7.5 ± 1.2	11.8 ± 1.8	15.3 ± 1.7	<0.001***
Surface roughness (μm)	0.6 ± 0.15	0.28 ± 0.04	0.85 ± 0.15	0.015*

## Discussion

The mechanical properties of dental materials play a critical role in their performance and longevity in dental restorations [9]. The comprehensive evaluation of mechanical and esthetic properties of dental implant materials, namely, titanium, zirconia, and ceramic, provides valuable insights into their performance and potential clinical implications.

Based on the results of this study, it is evident that different materials exhibit varying mechanical properties, which can influence their suitability for specific applications. Titanium demonstrates the highest tensile strength, indicating its superior mechanical robustness compared to zirconia and ceramic. This aligns with a study by Bojko et al., which evaluated the static strength in axial tensile tests of titanium alloys, highlighting their high ultimate tensile strength [10]. Additionally, zirconia stands out with the highest modulus of elasticity, suggesting its potential for enhanced resistance to deformation. This is supported by Özdoğan and Duymuş, who found that grinding produced the highest hardness values for zirconia ceramics, emphasizing its resistance to indentation and wear [11]. Furthermore, in terms of fatigue resistance, titanium demonstrates superior durability, outperforming both zirconia and ceramic. This is consistent with the findings of Thoma et al. who reported that after a loading period of six months, one-piece titanium dental implants render similar peri-implant soft tissue dimensions compared to zirconia implants [12].

Moreover, zirconia exhibits the highest hardness, emphasizing its resistance to indentation and wear. This is further supported by a study by Zidan et al. which documented that the surface hardness of polymethyl methacrylate (PMMA) with zirconia nanoparticles significantly improved compared to the nanocomposite group containing silanised zirconia [13]. Additionally, zirconia's potential for enhanced mechanical properties is highlighted in a study by Zidan et al., which found that reinforcement of conventional denture base resins with zirconia nanoparticles significantly improved mechanical properties such as flexural and impact strength, as well as surface hardness [14].

In contrast, the mechanical properties of titanium can be further enhanced through surface treatments. Shot peening has been shown to improve the fatigue performance and failure mechanisms of titanium specimens, indicating its potential to enhance the material's mechanical properties [15]. Furthermore, the experimental study by Tikhilov et al. confirmed the possibility of achieving a strong attachment to the bone with a tensile strength of 148 N for porous titanium implants, highlighting the potential for further improving the mechanical properties of titanium through innovative designs and treatments [16].

In summary, the mechanical properties of titanium, zirconia, and ceramic materials play a crucial role in determining their suitability for various applications. While titanium exhibits superior tensile strength and fatigue resistance, zirconia demonstrates high hardness and modulus of elasticity. These properties can be further enhanced through surface treatments and reinforcements, offering opportunities to optimize the mechanical performance of these materials for specific applications.

The findings of this study indicate that zirconia exhibits favorable characteristics in terms of color stability, translucency, and surface roughness compared to titanium and ceramic materials. Zirconia emerged as a frontrunner in color stability, boasting the lowest ΔE value, suggesting its superior resistance to staining agents compared to titanium and ceramic. This is supported by Alzanbaqi et al. who provided detailed findings about the color stability of zirconia crowns, emphasizing its potential for maintaining color integrity [17]. Furthermore, the translucency analysis reveals that ceramic offers the highest translucency, potentially making it a preferable choice for achieving natural-looking restorations. This aligns with the study by Tabatabaian, which discussed potent factors in the color of monolithic zirconia restorations, highlighting the impact of material reduction on color stability [18].

Moreover, zirconia exhibits the lowest surface roughness, indicating a smoother surface that may contribute to improved soft tissue compatibility. This is consistent with the study by Ganbold et al. which compared the surface roughness of zirconia and titanium specimens, highlighting the smooth surfaces of zirconia [19]. Additionally, the optical properties of translucent zirconia are affected by factors such as crystal phase, morphology, and flexural strength, indicating the multifaceted nature of zirconia's translucency [20]. Furthermore, the potential of highly translucent zirconia for improved esthetic appearance is highlighted in the study by Mao et al. emphasizing its attractiveness for dental applications [21].

Contrastingly, the color stability of zirconia may be influenced by factors such as immersion in beverages and exposure to oral conditions, suggesting the need for further studies to comprehensively assess its performance in different environments [22,23]. Additionally, the translucency of zirconia is affected by factors such as sintering parameters and accelerated aging, indicating the need for careful consideration of fabrication and environmental factors when evaluating its optical properties [24,25].

In summary, the findings demonstrate the favorable color stability, translucency, and surface roughness of zirconia compared to titanium and ceramic materials [26]. However, the performance of zirconia is influenced by various factors, necessitating further research to comprehensively understand its behavior in different conditions and applications.

The study's findings contribute valuable information to the evolving landscape of dental implant materials. While titanium remains a stalwart choice for its exceptional mechanical properties, zirconia emerges as a strong contender, particularly in cases prioritizing esthetics and fracture resistance. Ceramic, with its high translucency, may find a niche in anterior restorations where mimicking natural tooth appearance is crucial. Clinicians should weigh these material-specific properties against patient-specific needs and preferences, emphasizing a tailored approach in implant selection.

Future research could explore the in vivo performance of these materials, considering factors like bone integration, soft tissue response, and overall clinical success. Additionally, ongoing advancements in material science may introduce novel implant materials, warranting continuous exploration and comparison.

## Conclusions

This study provides a nuanced understanding of the mechanical and esthetic properties of titanium, zirconia, and ceramic dental implants. The results underscore the importance of considering material-specific characteristics in aligning implant selection with patient needs and treatment goals. As dental implantology continues to evolve, a thoughtful and evidence-based approach to material selection will play a pivotal role in achieving optimal clinical outcomes and patient satisfaction.
